# Impact of Insulin Degludec/Liraglutide Fixed Combination on the Gut Microbiomes of Elderly Patients With Type 2 Diabetes: Results From A Subanalysis of A Small Non-Randomised Single Arm Study

**DOI:** 10.14336/AD.2023.0118

**Published:** 2023-04-01

**Authors:** Stefano Rizza, Daniele Pietrucci, Susanna Longo, Rossella Menghini, Adelaide Teofani, Giacomo Piciucchi, Martina Montagna, Massimo Federici

**Affiliations:** ^1^Department of Systems Medicine and; ^2^Department of Biology, University of Rome Tor Vergata, Italy

**Keywords:** Liraglutide, aging, Type 2 diabetes, microbiota, GLP-1 RA

## Abstract

In elderly Type 2 Diabetes (T2D) patients the relationship between the destabilization of gut microbiome and reversal of dysbiosis via glucose lowering drugs has not been explored. We investigated the effect of 6 months therapy with a fixed combination of Liraglutide and Degludec on the composition of the gut microbiome and its relationship with Quality of Life, glucose metabolism, depression, cognitive function, and markers of inflammation in a group of very old T2D subjects (n=24, 5 women, 19 men, mean age=82 years). While we observed no significant differences in microbiome biodiversity or community among study participants (N = 24, 19 men, mean age 82 years) who responded with decreased HbA1c (n=13) *versus* those who did not (n=11), our results revealed a significant increase in Gram-negative *Alistipes* among the former group (*p*=0.013). Among the responders, changes in the *Alistipes* content were associated directly with cognitive improvement (r=0.545, *p*=0.062) and inversely with TNFα levels (r=-0.608, *p*=0.036). Our results suggest that this combination drug may have a significant impact on both gastrointestinal microbes and cognitive function in elderly T2D individuals.

## INTRODUCTION

Aging is a major non-reversible risk factor for type 2 diabetes mellitus (T2DM) [1]. Because diabetes management in older patients can be complicated by polypharmacy and cognitive impairment, we recently conducted an interventional pilot clinical study which resulted in broadly-perceived improvements in quality of life and cognitive function when we replaced complex anti-diabetic regimens with a single daily dose of fixed combination of insulin degludec and liraglutide (insulin degludec at 100 units/mL and the glucagon-like peptide 1 receptor agonist [GLP-1RA] liraglutide at 3.6 mg/mL) [2]. While results from several recent studies suggested GLP-1RAs might alter the gut microbiome [3], and others examined the impact of the gut microbiome and its role in modulating depression, cognitive impairment, and T2DM [4], no data are available that address the role(s) of these new agents and their specific impact on the gut microbiomes of elderly T2DM patients. Here we report the results of a metagenomics analysis of gut microbiota in elderly patients treated for six months with a fixed combination of insulin degludec and liraglutide.

## MATERIALS AND METHODS

The findings presented in this study are the result of a sub-analysis of an open, single-arm six-month interventional trial conducted in a real-world setting. All study procedures were performed in compliance with ethical standards for human clinical trials (institutional and national) and with the Declaration of Helsinki of 1964 (revised in 2013). This study was approved by the University Hospital Committee of “Tor Vergata” University (protocol number: 141/18) and registered at ClinicalTrials.gov ID: NCT04190160. Informed consent was obtained before screening and every procedure performed during the protocol. The study parameters were described extensively in a previous publication [2]. The original study included 35 patients (12 women, 23 men, mean age, 81.4 years) who replaced their pre-existing hypoglycemic therapeutic regimen with a flexibly-timed single daily dose of degludeg and liraglutide fixed combination. The clinical protocol included two ambulatory visits, including one at the beginning of the study (V0) and another six months after changing to degludeg and liraglutide combination (V1). Fecal samples collected at each visit were divided into aliquots and stored at -80 ?C until analysis.

### Assesment of Quality of Life, cognition, depression, and level of independence

We used Control, Autonomy, Self-Realization, and Pleasure-19 (CASP-19) scale to explore factors that affect QoL at an older age and the Diabetes Treatment Satisfaction Questionnaire (DTSQ) to evaluate the self-reported satisfaction related to a change in diabetes therapy. We also used the Geriatric Depression Scale (GDS) to assess depression symptoms whereas Mini Mental State Examination (MMSE) were used to screen the cognitive function in our study population; change in level of independence was assessed by activities of daily living (ADL) and by instrumental activities of daily living (IADL).

### DNA extraction and 16S rRNA gene sequencing

Total DNA was extracted from 200 mg of each frozen stool sample using the PSP Spin Stool DNA Kit (Stratec Molecular, Berlin, Germany), following the manufacturer's protocol. Briefly, stool samples were lysed under denaturing conditions at 95 °C and then treated with proteinase K at 70 °C. DNA was purified through a spin column system, eluted, and quantified using a NanoDrop spectrophotometer ND1000 (ThermoFisher). Sequencing of 16S rRNA amplicons (V3-V4 regions) was performed using Illumina MiSeq 2x300bp.

**Table 1:** Patient characteristics at baseline (V0) and after six months on Degludec and Liraglutide.

	Degludec and Liraglutide responders (n=13)			Degludec and Liraglutide non-responders (n=11)			
*Variables*	V0	V1	*p	V0	V1	**p	p***
Age (y)	82 ± 4.6		*n.p.*	82.3 ± 4.7		*n.p.*	*0.871*
Sex (m/f)	10/3		*n.p.*	9/2		*n.p.*	*0.768*
BMI (kg/m^2^)	28.8 ± 3.8	28.0 ± 3..4	0.070	29.1 ± 3.8	28.0 ± 3.3	0.001	
Systolic BP (mmHg)	124.0 ± 11.0	127.6 ± 22.3	0.511	136.4 ± 20.1	134.8 ± 19.1	0.501	0.055
Diastolic BP (mmHg)	76.7 ± 7.2	69.5 ± 6.7	0.022	72.0 ± 8.8	72.7 ± 9.4	0.775	0.130
Fasting glucose (ng/dl)	193.6 ± 70.2	129.8 ± 27.1	0.007	153.0 ± 34.2	127.6 ± 38.4	0.028	0.059
HbA1c (%, mmol/mol)	8.07 ± 1.02, 65.1 ± 12	6.60 ± 0.5, 49.0 ± 07	0.001	7.35 ± 0.82, 57.2 ± 10	7.72 ± 1.0, 61.4 ± 12	0.009	0.047
Total cholesterol (mg/dl)	152.4 ± 32.3	138.8 ± 27.1	0.044	181.1 ± 28.3	168.1 ± 30.5	0.249	0.021
Creatinine (mg/dl)	1.14 ± 0.29	1.09 ± 0.28	0.755	1.11 ± 0.62	1.18 ± 0.34	0.519	0.812
e-GFR (ml/min/1.73 m^2^)	41.32 ± 5.3	43.55 ± 5.1	0.322	39.2 ± 7.3	38.71 ± 6.8	0.666	0.138
IL-1β (ng/ml)	0.367 ± 0.802	1.611 ± 5.16	0.684	0.165 ± 0.109	0.105 ± 0.130	0.057	0.218
IL-6 (ng/ml)	4.97 ± 4.58	4.80 ± 5.35	<0.001	7.15 ± 11.05	8.86 ± 14.17	0.229	0.441
TNFα (ng/ml)	15.37 ± 4.46	12.98 ± 4.122	0.002	16.58 ± 4.67	15.15 ± 2.91	0.227	0.347
MMSE	23.5 ± 2.8	24.7 ± 3.3	0.102	23.7 ± 3.5	24.5 ± 2.2	0.220	0.911
GDS	5.9 ± 4.4	4.5 ± 3.8	0.106	4.8 ± 2.6	3.7 ± 2.2	0.124	0.442
DTSQs	21.7 ± 10.0	35.9 ± 4.9	<0.001	27.4 ± 8.1	34.7 ± 3.9	0.009	0.107
CASP-19	45.4 ± 6.5	42.4 ± 5.2	0.126	39.8 ± 7.4	39.4 ± 6.2	0.524	0.053

*Footnotes:* Responders: study participants exhibiting reductions in HbA1c of 0.3% or more at V1 compared to their individual baseline readings (V0). Non-responders: study participants who did not exhibit reductions in HbA1c of 0.3% or more at V1 compared to their individual baseline readings (V0); *p values for V0 *vs.* V1 among the Degludec and Liraglutide responders; **p values (V0 *vs.* V1) for Degludec and Liraglutide non-responders.V0, baseline visit; V1, after six months of treatment with Degludec and Liraglutide; ***p values (V0 responders vs V0 non-respondrers; BMI, body mass index; BP, blood pressure; HbA1c, glycated hemoglobin; e-GFR, estimated-glomerular filtration rate; IL-1β, interleukin-1β; IL-6, interleukin-6; TNFα, tumor necrosis factor-α; MMSE, mini-mental state examination; GDS, geriatric depression scale; DTSQs, diabetes treatment satisfaction questionnaires; CASP-19, control, autonomy, self-realization and pleasure-19

### Bioinformatics and statistical analyses

Bioinformatics data were generated as previously described [5]. After completion of the quality checks, reads were clustered using DADA2 and taxonomically assigned using the Silva database vr. 138 in the QIIME2 (pipeline) [6, 7]. Metagenomic data were normalized and analyzed using the statistical approaches described by Cerroni et al. [8]. We searched for bacterial taxa with abundances that differed between groups using Repeated Measures ANOVA. This model can be used to identify differences in abundance that are potentially related to (1) degludeg and liraglutide combination treatment (i.e., “responder” *versus* “non-responder” with respect to reductions in HbA1c), (2) the time of the measurement, or (3) the combined effects of both treatment and time.

## RESULTS

Fecal samples were obtained at baseline (V0) and six months after switching to insulin degludec and liraglutide fixed combination (V1) from 24 of the original 35 study patients. Eleven patients were excluded from the metagenomic analysis because they did not collect sufficient feces samples in visit 2.

Patients exhibiting a 0.3% decrease in HbA1c at V1 compared to V0 were classified as responders (n=13); those who did not exhibit at least 0.3% reductions in HbA1c were classified as non-responders (n=11). Table 1 provides a detailed comparison of clinical and biochemical characteristics of study patients classified as either responders or non-responders as described above. Notably, depression and cognitive function did not show any significant modification in both groups during the six months protocol. Interestingly, DTSQs increased whereas TNFα levels decreased only in responders (Table 1)

After the deletion of low-quality and chimeric reads, amplicon sequence variant (ASV) clustering, and removal of low-abundant taxa, 555201 reads were available for statistical analysis. We identified 2731 ASVs representing 254 species, 193 genera, 53 families, 27 orders, 19 classes, and 12 phyla.

**Figure 1.**
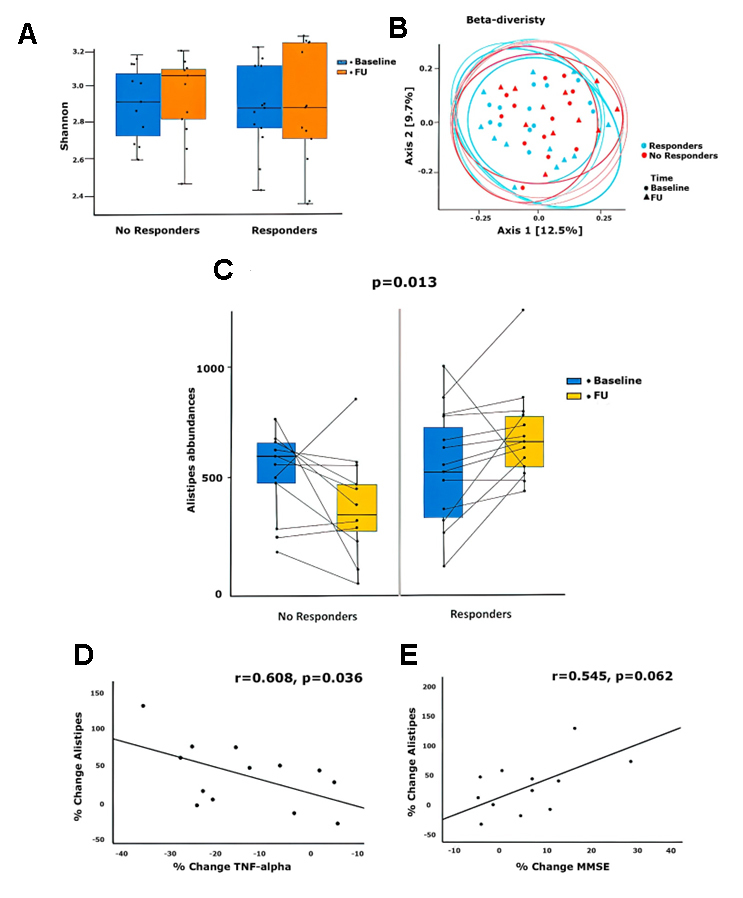
Changes in biodiversity (A) and community (B) of the microbiome composition in patients undergoing insulin degludec and liraglutide fixed combination treatment. (C) Variations in the abundance of Alistipes species in Responders compared to Non-responders to insulin degludec and liraglutide treatment: baseline (V0) to follow-up (V1); p-value=0.013. Correlation between percentage change in Alistipes and TNF-α levels (r = -0.608, p = 0.036) (D) and MMSE score (r = 0.545, p = 0.062) (E) among responders to insulin degludec and liraglutide.

We detected no significant differences at biodiversity and community levels in fecal samples from patients classified as responders and non-responders based on the Shannon index (Fig. 1A) or PERMANOVA (Fig. 1B) as measures of alpha and beta diversity, respectively. We then evaluated our findings with a repeated measure ANOVA to identify taxa whose abundances might differ between responders and non-responders over time. We excluded taxa that exhibited the same trend in each of the two groups as well as those that differed in a time-independent manner. Although this secondary analysis yielded no statistically significant findings, we identified one taxon that exhibited a particularly interesting trend. Specifically, our results revealed an increase in the proportion of bacteria of the *Alistipes* genus in samples from the responders, with a mean number of normalized reads at the baseline (V0) of 468 and a mean number of normalized reads after six weeks of degludeg and liraglutide fixed combination (V1) of 618. By contrast, we observed a decrease in the proportion represented by this genus in samples from patients categorized as non-responders, with a mean number of normalized reads of 477 and 438 at V0 and V1, respectively. We interpreted this result as a trend because the *p*-value was significant only before the correction for false discovery rate (FDR) was applied (*p*=0.013, without FDR correction) (Fig. 1C). Of note, we observed no significant modification in cognitive function explored by MMSE in our participants either in non-responders or in responders (Table 1).

Finally, we generated a correlation matrix to identify any associations between changes observed in the prevalence of this genus and clinical parameters. Linear correlation analysis revealed that increased abundances of *Alistipes* were directly associated with percentage increases in MMSE scores (r=0.43, FDR=0.0027). Only in responders we also observed a statistically significant inverse association between the percentage change in *Alistipes* abundance and the percentage change in serum TNFα levels (r=-0.608, p=0.036; Fig. 1D) and a strong, albeit not a statistically significant, direct association between percentage change in the abundance of *Alistipes* and percentage change in MMSE scores (r=0.545, *p*=0.062, Fig. 1E). On contrast, we did not observe any significant correlation between the change in *Alistipes* abundance and serum IL-1β and IL6 levels.

## CONCLUSIONS

Several publications have described the critical contributions of the intestinal microbiome to the health and well-being of elderly individuals largely based on its capacity to influence metabolic and digestive functions, as well as depression, cognitive impairment, immunity, and resistance to infections [9-10]. However, the mechanisms underlying these positive responses remain unclear.

The results of the present study revealed that six months of fixed combination of insulin degludec and liraglutide did not affect depression and cognitive function assessed by GDS and MMSE, respectively. Similarly, the drug combination had no impact on the composition of the gut microbiomes of elderly T2DM patients that responded with reductions in HbA1c (i.e., responders). This last fact suggested that drug-dependent changes in serum glucose levels had no direct impact on the gut microbiomes of these patients. Interestingly, our analysis revealed an increase in the percentage of bacteria of the *Alistipes* genus among patients identified as responders. Although this finding was interpreted as a trend because statistical significance was lost after the application of an FDR correction, this may be due in part to the low number of samples analyzed in this study. Thus, we believe that this trend could be of clinical interest and might be evaluated further in future studies.

*Alistipes* are anaerobic Gram-negative bacteria of the phylum, Bacteroidetes. While these bacteria are commonly found in healthy human intestinal microbiota, recent studies have identified alterations in the abundance of *Alistipes* in patients with non-communicable disorders, including liver diseases, colorectal cancer, atherosclerosis, and mood disorders [11]. Of particular interest, one recent publication [12] reported that Japanese centenarians exhibited a pronounced abundance of fecal *Alistipes* compared to younger controls; the authors proposed this finding as a potential marker of successful aging. However, we recognize that our study patients were from a Caucasian population; previous studies have yielded conflicting insights into the implications of changes to the gut microbiome-based specifically on ethnicity [13].

*Alistipes* produce and release acetate in the form of short-chain fatty acids (SFCAs) [14] that may serve to maintain hepatic energy balance by controlling appetite and mediating glucose homeostasis at the systemic level [15]. Moreover, acetate may limit lipopolysaccharide-induced TNFα secretion via stimulation of free fatty acid receptors on mononuclear cells [16]. Although we cannot exclude potential contributions from an intermediary mechanism, the observed increase in the abundance of *Alistipes* may lead to a parallel increase in SCFA concentrations and ultimately reductions in neuroinflammation. Consistent with this hypothesis, we observed that increases in the proportion of *Alistipes* in fecal samples were associated directly with cognitive improvement. Moreover, although the was no association between TNFα levels and the increasing of Alistipes abundances, the entanglement between Alistipes modulation and neuro-inflammation needs to be further investigated (Fig. 1D and E).

It has well known that various drugs could impact on gut microbiomes [17]. In this context, metformin may influence the altered gut microbiota affecting specific pathways such as metalloproteins or metal transporters functions [18]. However, in our study the beneficial effects of insulin degludec [19] and liraglutide [20], both associated with a reduced risk of dementia in frail, older adults with T2DM, may have been blunted in non-responders, potentially due to dysbiosis and/or drug resistance. Similarly, while our findings suggest that the fixed combination of Degludec and Liraglutide might have an impact on the relative distribution of microbes in the gastrointestinal tract microbes in elderly T2DM patients, our study involved only a limited number of subjects. Our hypothesis-generating results will need to be validated in a larger study of patients with similar characteristics.
